# National Physical Training

**Published:** 1920-03-06

**Authors:** 


### NATIONAL PHYSICAL TRAINING.

### The Outline of a New Scheme.

At a recent meeting of the Royal Society of Medicine Surgeon-Commander K. Digby Bell, R.N., of the Royal Naval School of Physical and Recreational Training, made an eloquent and convincing appeal to the medical profession to give their earnest consideration to the matter of physical training.

During the war many valuable lessons have been learned in connection with the subject. Up North, Dr. Bell said, the spectre to be considered seriously was the possible onset of a depression due to the incessant nervous strain and ghastly monotony. One lesson learned was that recreation and more recreation of a simple and attractive nature for *all* on board ship was wanted. It was always possible to keep the 10 per cent. who excelled at games amused and energetic. They would roller-skate, box, dance, or do anything to keep themselves fit and cheery when weather and other conditions made landing on shore impossible. But it was the 90 per cent. who were not endowed with this natural ability on whom they had to concentrate in order to kindle a small spark of energy to shake off the dull and listless mood into which they were unconsciously drifting. Recreational committees were formed and schemes worked out to assist those ships less fortunately equipped and situated. Physical training began to be looked upon in a much bigger light; the study of amusements of a healthy and stimulating nature for the whole community was embodied in it; the need for that psychological element of personal touch between officer and man was never more apparent than at such a time as this. So, when the Physical Training-school was revived at Portsmouth early last year, the subject had to be reviewed afresh.

This element of recreation had also been realised as an integral part of the scheme of training in our armies at home and abroad. That exceptionally fine system of treating the trench-weary soldier had been inaugurated by Colonel R. Campbell, D.S.O., at his headquarters of P. and B. Training at St. Pol. Consequently it had to be realised that physical education embodied a larger field of study and research now than was ever believed in pre-war days.

In the scheme which has been elaborated by Commander B. T. Coote, the technical adviser to the Royal Naval School of Physical and Recreational Training, and which is now in actual use there in the training of the officers now undergoing instruction, the objects of physical training are considered under three main headings. These are:—Activity, developed by physical training; energy, developed by recreational training; and character, developed by moral training.

### Physical Training.

Under the title of activity are included methods which are Preventive, Constructive, Continuative, Corrective, and Destructive.

#### *Preventive Measures.*

These are dealt with by lectures on the anatomical and physiological hygiene of the body, pointing out those elements of daily existence which are harmful to the development of a healthy and active frame. Anatomy and physiology naturally come prominently into this heading.

#### *Constructive Measures.*

These speak for themselves. Here is the science of applying bodily exercise to the young which will ensure as perfect and fit a body as it is possible to acquire. This is dealt with under the following headings:—

(*a*) *Physical Drill.*—This may be defined as the application of any bodily movement to produce a spectacular effect rather than a constructive effect. An instructor can apply drill, but a teacher works for the constructive effect on the bodies and minds of those he is training.

(*b*) *Controlled Exercises* embody the minute study of the application of the Swedish system of gymnastics, made attractive so as to suit the characteristics of our nation.

(*c*) *Controlled Games* consist of a large series of those games, taught in the uncontrolled stage under recreational training, which a teacher may wish to employ in his gymnastic table to combine constructive exercise with attractiveness.

(*d*) *Applied Subjects* are the connecting-link between physical and recreational training, but more easily dealt with under the former heading.

#### *Continuative Measures.*

No system of education which does not ensure continuity should be worthy of consideration. It is necessary to understand the difference between what may be called educative physical training, that is to say, the definite constructive development of the bodies of young and growing people, and the maintenance of physical fitness in the fully developed bodies of those of maturer age. There comes to the latter, sooner or later, a desire for physical improvement, and with it a conviction that some form of daily exercise is necessary to ensure bodily health and activity. Activity exercises have been compiled for this purpose. They are simple in character. They take two minutes to do. They provide full and free movement of the body in an interesting and attractive form.

#### *Corrective Measures.*

Orthopædics is a subject which at once calls for the knowledge and assistance of the medical profession. When it is realised that the following percentages of abnormalities were found to exist, before the war, in certain scholastic establishments selected as a test, it will be readily under-

**National Physical Education—(*continued*).**

stood how necessary it is for this subject to be seriously considered:

| | Per cent. |
|---|---|
| Flat feet ... ... ... ... ... ... | 33 |
| Weak ankles ... ... ... ... ... | 33 |
| Knock knees ... ... ... ... ... | 33 |
| Chest malformations ... ... ... | 63 |
| Spinal irregularities ... ... ... | 15 |

*Destructive Measures.*

A teacher of physical education should know what evil effects may accrue from an injudicious use of bodily activities, especially at such times as these, when our papers are filled with advertisements of so-called physical-culture experts. This subject is dealt with by lectures and practical demonstrations.

Recreational Training.

Here we have a more than essential element in the scheme of physical education; it is the very foundation of such a science. It provides measures for dealing with all types of pure recreation, and must be applicable to the majority rather than the minority. We all know the inestimable value of the sports and games we were fortunate enough to be taught, and get facilities to participate in, in our school and 'Varsity days. How they literally moulded our character and taught us, almost unconsciously, to play the game for our side, and not for self; how foul play was penalised; how energy and honour were essential to success. But what of the masses who were not so fortunate—those who had not the opportunities of obtaining the variety of childish games which teach the importance of obeying rules; whose football and cricket consisted more often of noisy squabbles and fighting in the streets and squares in their neighbourhood; whose only chance of learning something of our so-called national sports was by perhaps being lucky enough to attend a football match where twenty-two paid players performed before thousands and thousands of onlookers; or, perhaps, it may be a boxing contest in some hall, where anything but clean sport was exhibited to their enquiring minds? Think what chance these millions have had of learning what is meant by playing for their side, for their native town, their country. What chance have they had of moulding their characters on fine lines?

Let us think what this means to a nation. Recreation develops energy. We must conclude that energy is dwindling because more people are willing to watch than to take part, and recreation does not attract. To put it in another way, 10 per cent. wish to play and 90 per cent. are content without.

Say it is agreed that 10 per cent. of the population are born to excel at sports and games. In the past the needs of these have been specially catered for, and obviously will continue to be, no doubt. The 90 per cent. have never indulged systematically in sports and games, principally because they have not had the opportunity; or perhaps they have been discouraged by their own lack of ability to compete with the 10 per cent.

This scheme of recreational training applies principally to the 90 per cent. (otherwise it would not be a true educational scheme), and provides for them:—

1. An immense range of physical and recreational activities.
2. A new and hitherto undeveloped incentive to enjoy them.
3. An organisation whereby these may be applied in such a way as to secure the utmost advantage as regards the development of *energy* and *character*.

Moral Training.

Herbert Spencer wrote, "Of the ends to be kept in view by the legislator, all are unimportant compared with the end of character making—this alone is National Education." The development of character permeates the whole working of this scheme of physical education. Each individual is taught to respect himself and others. Patriotism and discipine are developed in a simple and attractive way.

Lectures on psychology and on the psychology of teaching are given. The attempt to interest the individual in his own existence is made by lectures, assisted by the microscope, magic-lantern, and specimens which deal with the simple truths of cell life, of botany, and of reproduction. The human body is built up in a simple and useful way, so as to enable the teacher to understand the why and wherefore of physical and recreational training. Further, the sex question is developed at some length; puberty and adolescence in the boy is dealt with, along with the moral and sexual difficulties of young adult life.

*Discipline* is a subject of the utmost importance in these days. It must be dealt with on the lines of *esprit de corps* and interest, as opposed to a discipline by fear of punishment, as far as the young idea is concerned. So important has this reconciliation of discipline become that, in future, if war ever has to come again, the very survival of democracy may hang on the solution of this problem to-day.

The Game of Life.

This title embodies the whole of the ideals of the system. The rules are few and simple. They are these: Don't play foul; don't chuck up the sponge; go all out to win; play for your side, not for yourself.

In conclusion Dr. Bell put forward the suggestion that the Government should be approached by a strong appeal from the medical profession of Great Britain for recognition of the subject as a large branch of preventive medicine by:—

(i) The formation of a National Council of Physical Education, to study and elaborate the lines our national system of physical education should take;

(ii) The formation of a British Physical Educational Association on some such lines as our present British Medical Association, to control all members practising physical education in Great Britain;

(iii) The formation of a Central Gymnastic Institute, where teachers of physical education could be educated on such lines as would be directed by the National Council and obtain the certificate of the association mentioned above.

